# Identification of Small Molecule Lead Compounds for Visceral Leishmaniasis Using a Novel Ex Vivo Splenic Explant Model System

**DOI:** 10.1371/journal.pntd.0000962

**Published:** 2011-02-15

**Authors:** Yaneth Osorio, Bruno L. Travi, Adam R. Renslo, Alex G. Peniche, Peter C. Melby

**Affiliations:** 1 Department of Veterans Affairs Medical Center, Research Service, South Texas Veterans Health Care System, San Antonio, Texas, United States of America; 2 Department of Medicine, The University of Texas Health Science Center, San Antonio, Texas, United States of America; 3 Small Molecule Discovery Center, Sandler Center for Basic Research in Parasitic Diseases, and Department of Pharmaceutical Chemistry, University of California San Francisco, San Francisco, California, United States of America; 4 Department of Microbiology and Immunology, The University of Texas Health Science Center, San Antonio, Texas, United States of America; McGill University, Canada

## Abstract

**Background:**

New drugs are needed to treat visceral leishmaniasis (VL) because the current therapies are toxic, expensive, and parasite resistance may weaken drug efficacy. We established a novel ex vivo splenic explant culture system from hamsters infected with luciferase-transfected *Leishmania donovani* to screen chemical compounds for anti-leishmanial activity.

**Methodology/Principal Findings:**

This model has advantages over in vitro systems in that it: 1) includes the whole cellular population involved in the host-parasite interaction; 2) is initiated at a stage of infection when the immunosuppressive mechanisms that lead to progressive VL are evident; 3) involves the intracellular form of *Leishmania*; 4) supports parasite replication that can be easily quantified by detection of parasite-expressed luciferase; 5) is adaptable to a high-throughput screening format; and 6) can be used to identify compounds that have both direct and indirect anti-parasitic activity. The assay showed excellent discrimination between positive (amphotericin B) and negative (vehicle) controls with a Z' Factor >0.8. A duplicate screen of 4 chemical libraries containing 4,035 compounds identified 202 hits (5.0%) with a *Z* score of <–1.96 (*p*<0.05). Eighty-four (2.1%) of the hits were classified as lead compounds based on the in vitro therapeutic index (ratio of the compound concentration causing 50% cytotoxicity in the HepG_2_ cell line to the concentration that caused 50% reduction in the parasite load). Sixty-nine (82%) of the lead compounds were previously unknown to have anti-leishmanial activity. The most frequently identified lead compounds were classified as quinoline-containing compounds (14%), alkaloids (10%), aromatics (11%), terpenes (8%), phenothiazines (7%) and furans (5%).

**Conclusions/Significance:**

The ex vivo splenic explant model provides a powerful approach to identify new compounds active against *L. donovani* within the pathophysiologic environment of the infected spleen. Further in vivo evaluation and chemical optimization of these lead compounds may generate new candidates for preclinical studies of treatment for VL.

## Author Summary

Visceral leishmaniasis is a life threatening parasitic disease present in several countries of the world. New drugs are needed to treat this disease because treatments are becoming increasingly ineffective. We established a novel system to screen for new anti-leishmanial compounds that utilizes spleen cells from hamsters infected with the parasite *Leishmania donovani*. The parasite strain we used was genetically engineered to emit light by the incorporation of the firefly luciferase gen. This laboratory test system has the advantage of reproducing the cellular environment where the drug has to combat the infection. The efficacy of the compounds is easily determined by measuring the light emitted by the surviving parasites in a luminometer after exposing the infected cells to the test compounds. The screening of more than 4,000 molecules showed that 84 (2.1%) of them showed anti-leishmanial activity and had an acceptable toxicity evaluation. Eighty two percent of these molecules, which had varied chemical structures, were previously unknown to have anti-leishmanial activity. Further studies in animals of these new chemical entities may identify drug candidates for the treatment of visceral leishmaniasis.

## Introduction

New drugs are desperately needed to treat visceral leishmaniasis (VL), and this in turn requires new approaches to discover novel lead compounds that might populate a pipeline of new therapeutics for patients with VL. Current therapies for the leishmaniases are toxic, difficult to deliver, expensive, and their efficacy is hindered by parasite resistance (reviewed in [1]). The pentavalent antimony compounds, sodium stibogluconate and meglumine antimoniate, have been the mainstay of anti-leishmanial chemotherapy for more than 40 years. The recommended regimen involves prolonged and often repeated courses of drug administered by the intravenous or intramuscular routes. Cure rates of 80–100% were common in the 1990s, but have dropped off considerably because of parasite resistance [2]. Adverse effects of antimony therapy are multiple and often dose-limiting. Amphotericin B desoxycholate and the amphotericin lipid formulations are also used in the treatment of VL, and in many regions have replaced antimony as first-line therapy. The use of these drugs, however, is limited by their difficulty of administration, well-known risk of toxicity, and high cost. Parenteral treatment of VL with the aminoglycoside paromomycin (aminosidine) is used in India but not licensed in the U.S. Miltefosine, a membrane targeting alkylphospholipid, was recently licensed in India as the first oral treatment for VL, but after only a few years of use, drug resistance has emerged. The discovery of new drugs for VL would have huge impact on individual patients and on populations in the endemic area as a whole.

Pre-clinical *in vitro* studies to identify candidate drugs for treatment of leishmaniasis have employed several different approaches, each of which has significant limitations. The testing of drugs using axenically cultured parasites, usually promastigotes, has been most commonly used, but this approach is limited by 1) the discordance of anti-leishmanial activity of compounds tested in axenically cultured promastigotes (vector stage) and amastigotes (mammalian stage) [3], [4], and 2) the testing of antiparasitic activity in the absence of host immune cells, which are known to profoundly influence parasite replication or killing [5], [6], [7], [8]. The use of cultured macrophages that have been infected in vitro with *Leishmania* has the advantage of identifying drugs that are active against the intracellular amastigote, but in vitro infections are technically cumbersome, may have a variable number of promastigotes that remain attached but not internalized by macrophages, and the isolated macrophage-amastigote infection excludes other immunomodulatory host cells such as disease promoting or regulatory T cells (reviewed in [9]).

Pre-clinical in vivo studies have largely used murine models of *Leishmania* infection, however, *L. donovani* infection in mice does not fully represent the features of active VL, and thus arrest or cure of severe, progressive disease and death cannot be an endpoint in therapeutic trials in mice. Strikingly, Syrian hamsters recapitulate the progressive clinicopathological features of human VL [10], [11], [12], most notably the profound immunosuppression associated with active disease that has fundamental importance to effective therapy. During progressive disease in the hamster model of VL, a Type 1 T cell response is mounted, but paradoxically it is ineffective [11], [12]. This contrasts sharply with the mouse model, but is very similar to what has been demonstrated in humans with VL [13], [14], who also mount an ineffective Type 1 response. Most notably, progressive disease in hamsters is accompanied by low NOS2 expression [11], and infected hamster macrophages, akin to human macrophages [15], [16] produce very low levels of NO [17]. In striking contrast, activated mouse macrophages produce high levels of NO. Thus, the macrophage defense against intracellular pathogens in hamsters is uniquely similar to what is observed in human macrophages.

Because of the profound influence of the host immune response on the treatment of *Leishmania* infection we sought to develop a test system that included the immunopathological milieu found at the site of the host-parasite interaction and active disease. This would enable the activity of new compounds to be determined within the context of the pathogenic mechanisms that contribute to progressive disease. To accomplish this, an *ex vivo* explant culture of spleen cells from *L. donovani* infected hamsters was established from hamster spleens at a point in the course of infection (day 21 post-infection) when disease and parasite replication are dramatically increased. This transition to explosive parasite replication and progressive disease is accompanied by a loss of T cell responsiveness (as occurs in human disease) and the development of an alternatively activated macrophage phenotype. The use of a parasite strain that expressed luciferase enabled determination of parasite killing by a large number of compounds in a medium- to high-throughput format. This approach enabled the identification anti-leishmanial drug candidates that are active in the face of the disease-promoting immune response, as must occur in the treatment of human VL.

## Materials and Methods

### Animals and parasites

Female inbred Chester Beatty hamsters (6–8 weeks-old) derived from our own breeding colony were used. *Leishmania donovani* (MHOM/SD/001S-2D) promastigotes were cultured in complete M199 (0.12 mM adenine, 0.0005% Hemin, 20% FBS) as described previously [18]. The *L. donovani* strain was transfected with an episomal vector containing the luciferase (luc) reporter gene [19] and was maintained routinely by isolation from infected hamsters, selection in complete M199 with 10 µg/ml of G418, and intracardial subinoculation of new hamsters approximately every 3 months. These studies were reviewed and approved by the Institutional Animal Care and Use Committee of the University of Texas Health Science Center.

### Development of the ex vivo splenic explant culture

Groups of 8 animals were infected by the intracardial route with 10^6^ purified metacyclic promastigotes [11] and the body and spleen weights recorded at 7, 14 and 21 days post-infection. At each time point, the splenocytes were harvested as described below and the number of cells and parasite burden determined by microscopy and luminometry, respectively. The luminometry counts were transformed to number of parasites using a linear standard curve of luciferase activity versus number of microscopically enumerated amastigotes. The amastigotes used for the standard curve were isolated from a cell suspension from hamster spleen at 20–30 days p.i. as follows: A splenocyte suspension was obtained by passing the spleen through a wire mesh and then the splenocytes were disrupted by passing sequentially through 27G½ and 30G½ needles, and polycarbonate membrane filters having pore sizes of 8 µm, 5 µm and 3 µm (Isopore, Millipore). Released amastigotes (free of host cells) were washed twice with PBS (3000 g ×10 min) and enumerated by direct microscopy. The alternative activation of macrophages was assessed by determining the arginase activity by the rate of urea formation from L-arginine in the presence of 1-phenyl-1,2-propanedione-2-oxime (ISPF) [20] and measuring the soluble collagen content (Sircol Assay, Biocolor Ltd). The cellular immune response was evaluated by the proliferative response of spleen cells to Concanavalin A as described [21].

### Cell populations in the ex vivo spleen explant culture

The general cell composition of the spleen explant was determined by microscopy and flow cytometry at 7, 14 and 21 days post-infection and compared with uninfected spleen cells. The spleen cells were suspended in DMEM +5% FBS at 10^6^ cells/100 µl, washed with PBS plus 0.1% BSA and 0.025% sodium azide, blocked for 20 min with PBS with 2% BSA and 5% of normal serum of the species in which the secondary antibodies were raised, and labeled with the monoclonal antibodies that are known to cross react with the corresponding hamster molecules. CD4 T cells were quantified by staining with rat anti-mouse CD4-PE (clone GK1.5; BD, 0.5 µg/tube) [22] followed by fixation, permeabilization (Leucoperm; Serotec), and labeling with rat anti-human CD3-FITC (CD3-12, Serotec, 0.5 µg per tube) which recognizes a highly conserved intracellular epitope of the CD3 molecule expressed by T lymphocytes in several species (manufacturer's data). B lymphocytes were quantified by labeling cells that expressed both the MHCII alloantigen (mouse anti-mouse I-E[k]-PE [clone 14-4-4S], BD, 0.5 µg/tube) [23] and IgG (Goat F(ab')2 anti-hamster IgG (clone Dlight 488; Serotec, 1 µg per tube). In all cases the percent of positive cells was determined by flow cytometry (FacsAria, BD) according to the threshold of the corresponding isotype controls.

### Parasite replication in the ex vivo spleen explant culture

The number of parasites and the proportion of infected macrophages were determined after 0, 24, 48 and 72 hours of ex vivo culture at 37°C, 5% C0_2_ using luminometry and light microscopy. The ability of the luminometric assay to discriminate between treated and untreated (control) splenic explant cultures was assessed in serial two-fold dilutions of cells cultured for 48 h in 100 µl of 0.2 µM amphotericin B (Sigma) or vehicle control.

### Splenic explant culture for drug screening and EC_50_ determinations

To establish the plate assay for drug screening, the spleens from 2 infected hamsters were aseptically removed and placed in a Petri dish containing 5 mL of Collagenase D (Roche) at 2 mg/mL of buffer (10 mM Hepes pH 7.4, 150 mM NaCl, 5 mM KCl, 1 mM MgCl_2_, 1.8 mM CaCl_2_). The spleen was infiltrated by injection with approximately 2 mL of Collagenase solution, the tissue was cut into small pieces using sharp scissors, and incubated for 20 minutes at 37°C. The cell suspension and remaining tissue fragments were gently passed through a 100 µm cell strainer (B–D) to obtain a single cell suspension, which was washed twice by centrifugation (500 g for 7 min at 4°C) and re-suspended in culture medium composed of DMEM (Gibco), 5% FBS, 1 mM Sodium pyruvate (Gibco), 1X MEM amino acids solution (Sigma), 0.02% v/v/EDTA, 10 mM HEPES buffer (Cellgro) and 100 IU/mL Penicillin/100 mg/mL Streptomycin solution (Cellgro). The splenocytes were counted and adjusted at concentrations from 100,000 to 500,000 cells/50 µL and used the same day for drug screening or determination of EC_50_ as described below.

### Primary screening of compound libraries using the ex vivo splenic explant model

The following chemical libraries in 96-well plate format were screened: *NINDS Custom Collection II* (MicroSource Discovery Systems, Inc.), which consists of 1,040 classical therapeutic agents, established experimental inhibitors, receptor agonist drugs and other bioactive compounds [24], [25], [26]; the *Pure Natural Products* library (MicroSource Discovery Systems, Inc.) a collection of 800 pure natural products and derivatives; and The Diversity Set and Natural Products set (Developmental Therapeutics Program, NCI/NIH) a set of 2,195 **c**ompounds selected from the almost 140,000 compounds based on diversity of structure [27], [28], [29]. Libraries containing the compounds at 10 mM concentration in DMSO were diluted into master plates at 200 µM concentration in DMEM. The 200 µM master plates were further diluted by transferring an aliquot of 5 µl into a 96-well sterile white-bottom plate (Costar) containing 45 µl of culture medium at 4°C. Splenocytes (100,000 in 50 µl of culture medium) that had been obtained from infected hamsters as described above were added to the assay plate for a final drug concentration of 10 µM. The positive control (splenocytes treated with 0.2 µM Amphotericin B) and negative controls (0.1% DMSO vehicle) were distributed in the two outer columns (8 wells each) of each plate in alternating fashion to minimize any edge effect [30]. After 48 hours of culture at 37°C in a humidified atmosphere and 5% CO_2_, the plates were centrifuged at 500 g for 7 min, the supernatant discarded using a multi- channel vacuum aspirator (Costar), and 20 µl of 1X cell culture lysis reagent (Promega) was added to the cells. To complete the lysis procedure the plates were frozen at −70°C and then thawed, and the luciferase activity determined in a plate luminometer following addition of 100 µl of the luciferin substrate at room temperature (Promega). The Z score was calculated for each compound as the mean counts of the compound tested minus the mean counts of all compounds in the plate divided by the SD all compounds in the plate [30]. The mean Z score for each drug was calculated based on duplicate screenings performed in two different experiments. A Z score of ≤–1.96, which corresponds to a *p* value of ≤0.05 (95% confidence limit), was used as the threshold to identify the hits [30]. The conditions of the screening were optimized to obtain the best signal to noise ratio by calculating the Z factor obtained after exposure to different drug concentrations (10, 5, 2.5 µM). The quality of each assay was determined by calculating the Z prime (Z') factor [31], which measures the discrimination between positive and negative controls in the screen. The Z' factor was calculated as 1 – [(3SD positive controls +3SD negative controls)/absolute value of (mean of the positive controls – mean of the negative controls)].

### Assessment of cell toxicity (CC_50_) using the HepG_2_ cell line

We used a cell-based assay as an alternative to animal testing to determine the toxicity of the identified hit compounds [32]. The cytotoxicity was evaluated in HepG_2_ cells (human hepatocellular carcinoma, ATCC#HB 8065) maintained in MEM (Gibco) supplemented with 10% FBS 1 mM sodium pyruvate (Gibco), 1X MEM aminoacids solution (Sigma), 100 IU/mL penicillin, and 100 mg/mL streptomycin (Cellgro). Cell monolayers were detached using 1X trypsin/EDTA (Gibco), washed and adjusted to 500,000 cells/mL in supplemented MEM, and 50 µl of the cell suspension were added to white-bottom 96-well plates (25,000 per well) containing 50 µl of serial 2-fold dilutions of the test compounds (0.1–100 µM) or the DMSO control. After 24 hours of culture at 37°C the number of viable cells was determined by quantification of the ATP present in the cell using the CellTiter-Glo luminescent Cell Viability Assay (Promega). The luminescence values were used to construct a curve using a linear regression model (GraphPad Prism 5.0) and calculate the cytotoxic concentration that killed 50% of the cells (CC_50_). The mean and standard error of 3–5 different experiments were considered as the final CC_50_ for the purpose of calculating the in vitro therapeutic index (see below).

### Anti-leishmanial efficacy (EC_50_) using the ex vivo splenic explant model

The anti-leishmanial efficacy of the compounds was determined using the splenic explant model in a 96-well plate format. In brief, splenocytes from infected hamsters were obtained as described above and a suspension of 100,000–500,000 in 50 µL of culture medium were added to white-bottom 96-well plates containing 50 µl of serial 2-fold dilutions of test compounds (0.03–20 µM) in culture medium. Because variation in the parasite burden between different explant cultures was expected, the concentration of cells used in each experiment was adjusted to give approximately 100 counts by luminometry (equivalent to ∼240,000 parasites). The number of surviving parasites was determined by luminometry as described above. Luminometry values were used to construct a curve using a linear regression model (GraphPad Prism 5.0) and calculate the effective concentration of the compound that killed 50% of the parasites (EC_50_). The mean and standard error of the EC_50_ from 2–3 different experiments were considered as the final EC_50_ for the purpose of ranking the compounds and calculating the in vitro therapeutic index (see below).

### Anti-leishmanial efficacy (EC_50_) using the in vitro infected macrophage model

Macrophages from uninfected hamsters were obtained by peritoneal lavage with DMEM (Gibco) and Heparin (2 units/ml; Elkins-Sinn, Cherry Hill NJ). The cells were washed twice by centrifugation and re-suspended in culture medium composed of DMEM, 5% FBS, 1 mM sodium pyruvate (Gibco), 1X MEM amino acids solution (Sigma), 0.02% v/v/EDTA and 10 mM HEPES buffer (Cellgro). The peritoneal macrophages were adjusted to 5×10^5^ cells/ml and allowed to adhere overnight at 37°C and 5% CO2 in flat bottom 96-well plates. Adherent macrophages were infected at 1∶5 ratio (cells:parasites) with stationary phase LUC-transfected *L. donovani* promastigotes and cultured at 37°C, 5% CO2, for 2 h. The extracellular parasites were then removed by washing with warm Dulbeco's PBS (Gibco). Infected macrophages were exposed to 2-fold serial dilutions of test compounds as described above for the ex vivo culture system. The infected macrophages were incubated for 48 hr in 5% CO_2_ atmosphere at 37°C. The number of surviving parasites was determined by luminometry and the effective concentration (EC_50_) calculated by linear regression (GraphPad Prism 5.0) as described above for the ex vivo system. The mean and standard error of the EC_50_ from 3 different experiments were considered as the final EC_50_. The test compounds Amphotericin B, Miltefosine, Pentamidine, Fluconazole, Antimycin A, Disulfiram, Monensin A and Nortriptyline were purchased from Sigma, and Meglumine antimoniate (Glucantime®) was obtained from Aventis Pharma (Brazil, Lot: L503451). Tilorone was obtained from Hangzhou Trylead Chemical Tech (China). All compounds were dissolved in sterile dimethyl sulfoxide (analytical grade, cell culture tested; Sigma) and stored frozen at −20°C until used.

### Identification of lead compounds

Hit compounds that showed a significant Z score in the ex vivo screening but demonstrated high toxicity for the HepG_2_ cell line (CC_50_ ≤10 µM) were excluded from further consideration. After the exclusion of these toxic compounds, the *in vitro* therapeutic index (IVTI), which is the ratio of the CC_50_ obtained in the HepG_2_ cell line and the anti-leishmanial activity (EC_50_), was calculated for each of the hit compounds. The resulting IVTI was used to rank the hits and select lead compounds that had an IVTI >5.

## Results

### Characterization of the ex vivo explant culture

**Figure 1:**
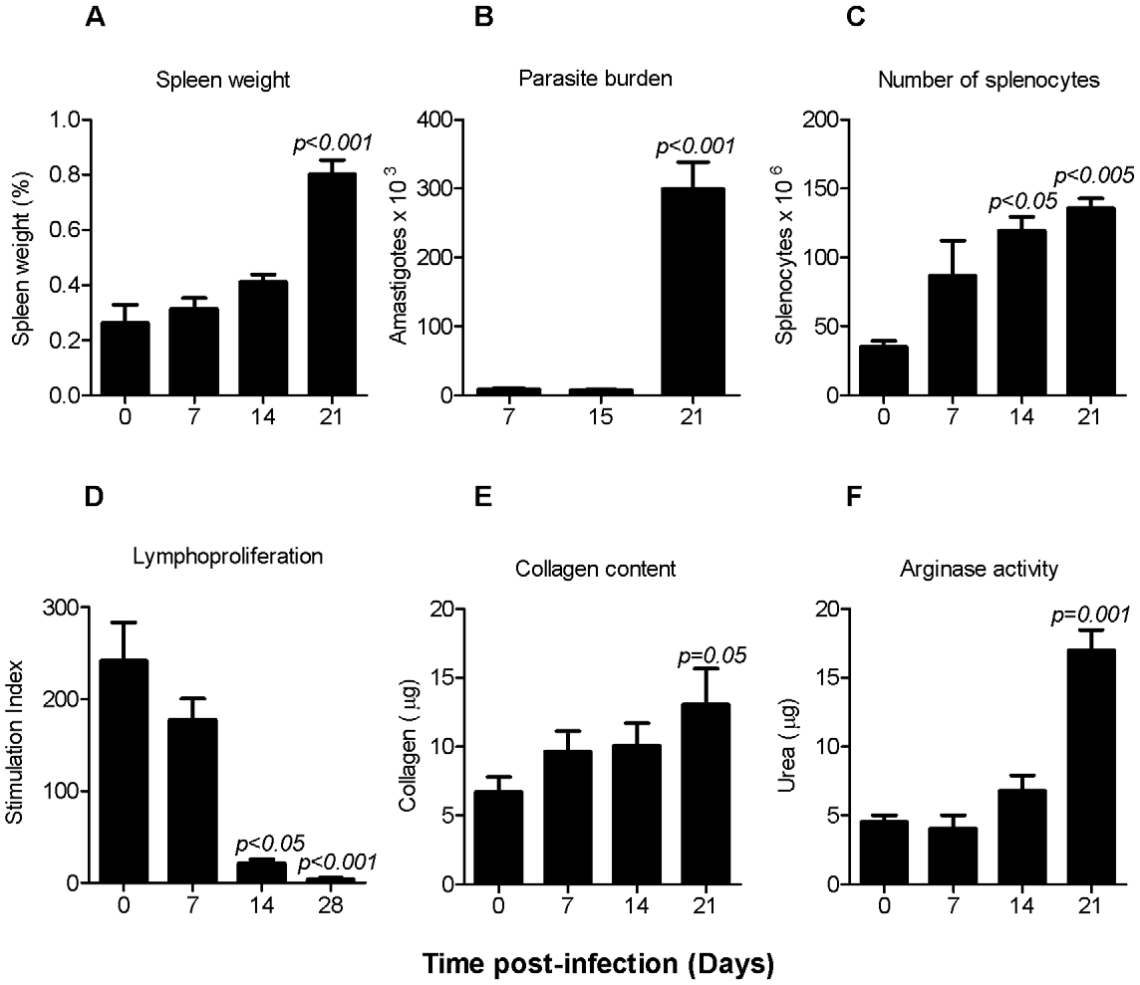
Rationale for selection of the time for establishing the ex vivo splenic explant culture. Hamsters infected with 10^6^ Luc-transfected *L. donovani* were evaluated from 7 to 21 days post infection (n = 6 per time point). **(A) Spleen weight.** Shown is the mean ± standard deviation (SD) of the spleen to body weight ratio (spleen weight divided the body weight). (B) **Splenic parasite burden.** The number of amastigotes (mean ± SD) was determined by luminometry in 500,000 splenocytes by extrapolating the counts (photons/sec) to a standard curve of microscopy-enumerated spleen-derived amastigotes. **(C) Total splenocyte number.** Splenocyte number (mean ± SD) was determined by counting the cells by microscopy. **(D) Splenocyte lymphoproliferative response.** The splenocyte stimulation index (shown as the mean ± SD) was determined by dividing the cpm of concanavalin A-stimulated and non-stimulated splenocytes. **(E) Splenic soluble collagen content.** The soluble collagen content (shown as the mean ± SD) was determined in spleens from uninfected and infected hamsters by the Sircol assay (Biocolor). **(F)** **Splenic Arginase activity.** Tissue arginase activity was determined by measurement of urea catalysis and is shown as the mean ± SD. Statistical analysis for all panels was performed by one-way analysis of variance (ANOVA).

We first characterized the clinical, immunological, and parasitological features of the infected hamster spleen early in the course of VL (which is ultimately fatal) to identify a time point that indicated the course of infection was transitioning toward severe disease. At 21 days p.i. there was a dramatic increase in spleen size (Fig. 1A), parasite burden (Fig 1B), and cellular infiltration of the spleen (Fig 1C). The splenomegaly was related to hypercellularity with a significant expansion of the macrophage population (6-fold increase over uninfected spleens, *p* = 0.016) and B lymphocytes (Table 1). The expansion of other cell populations that were not enumerated, such as fibroblasts, is likely to also contribute to the splenomegaly. The transition to progressive disease was also accompanied by loss of T cell responsiveness (Fig 1D) and preceded by a transient decrease in the percent of splenic CD3^+^ cells and CD4^+^ T cells at 14 days p.i. (Table 1). At 21 days p.i. there was also evidence of alternative macrophage activation with a significant increase in the amount of soluble collagen synthesis (Fig 1E) and arginase activity (Fig. 1F). Therefore, we selected day 21 p.i. as the time point to establish the ex vivo splenic explant culture so that the screening of small molecules for anti-*Leishmania* activity would be conducted within the milieu of the immunopathological mechanisms that lead to progressive VL.

**Table 1:** Cellular composition of the splenic explant culture from hamsters infected with *L. donovani*.

		Days post-infection 1			
Cell type	Result	0	7	14	21
Splenocytes 2	Total 2	35.0±10.8	86.6±56.6	119±23.3 c	135.8±16.0 b
CD3+ T Cells	% 3	21.6±5.4	23.3±3.7	13.6±3.5 c	18.5±0.5
	No. 4	7.6±1.9	20.2±3.2 d	16.1±4.2 d	25.1±0.7 d
CD4+ T Cells	%	5.6±1.9	6.9±3.5	2.6±0.5 c	6.1±0.9
	No.	1.8±0.7	6.0±3.0 b	3.1±0.5	8.3±1.3 a
B Lymphocytes	%	14.6±2.0	20.3±5.8 c	23.7±4.6 b	18.1±1.3
	No.	5.1±0.7	17.6±5.0	28.3±5.5 a	24.6±1.8 b
Macrophages	%	15.4±4.3	17.4±3.0	9.5±2.2	23.8±2.6 c
	No.	5.4±1.5	12.0±7.1	11.4±2.6	32.3±3.5 a
Granulocytes 5	%	3.15±1.2	0.96±0.4	1.44±0.2	0.46±0.1

1 Determined in 5 uninfected hamsters and 5 infected hamsters at each time point. The value represents the mean ± standard deviation of the group.

2 Number of total splenocytes (millions) obtained from the spleen of the animals. Enumerated in a Neubauer chamber by light microscopy.

3 Percentage of positive cells determined by flow cytometry.

4 Number of cells (millions): calculated from the total number of cells multiplied by the percentage of cells positive by flow cytometry.

5 Granulocytes: percentage of granulocytes determined by Side Scatter and Forward Scatter of cells by flow cytometry.

a p<0.001 uninfected vs. infected groups;

b p<0.01; uninfected vs. infected groups;

c p<0.05, uninfected vs. infected groups (Kruskal-Wallis test);

d p<0.001, uninfected vs. infected groups (Tukey-Kramer Multiple comparisons test).

### Optimization of the ex vivo splenic explant assay for drug screening

**Figure 2:**
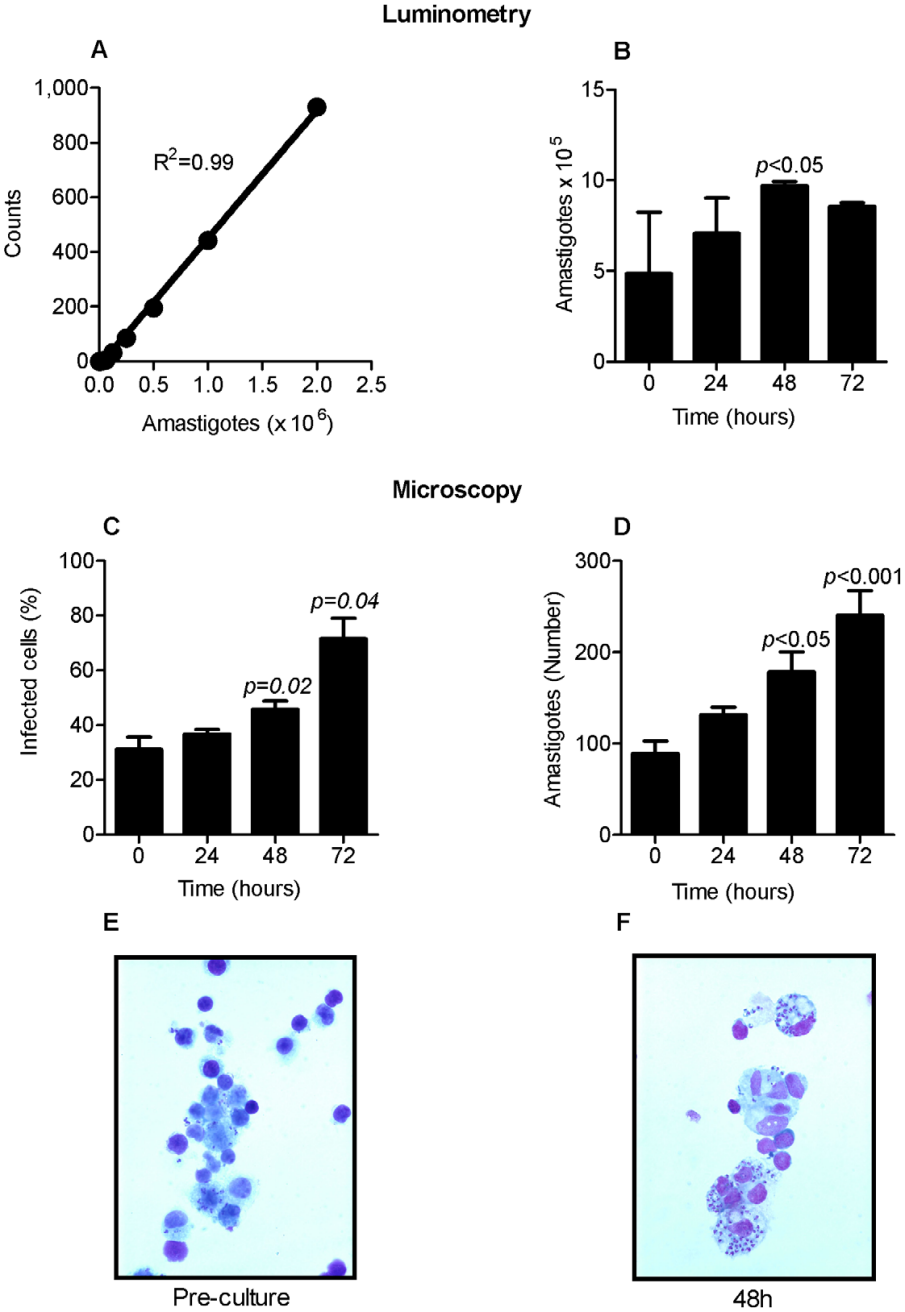
Characterization of the splenic explant cultures. **(A) Representative amastigote standard curve.** Correlation between the number of *L. donovani* amastigotes counted by microscopy and the luciferase activity determined by luminometry. **(B)** **Amastigote replication in splenic explant cultures.** Number of amastigotes in ex vivo explants cultures determined by luminometry and interpolation from the standard curve over 0 to 72 hours of incubation (100,000 splenocytes per well). **(C) Percent of infected macrophages in splenic ex vivo cultures.** Splenocytes harvested from infected hamsters (21 days p.i.) were plated and the proportion of infected macrophages (shown as the mean ± SD) was determined by microscopy at 0, 24, 48, and 72 hours of *ex vivo* culture. **(D)** **Number amastigotes per 100 macrophages**. Amastigotes enumerated by direct microscopy in Giemsa stained cytospin slides (mean ± SD in 4 different samples per time point). **(E, F) Infected splenocytes in** ***ex vivo*** **culture.** Representative Giemsa-stained photomicrograph of splenocytes from hamsters infected with *L. donovani* at pre-culture (E) and after 48h of *ex vivo* culture (F).

For the ex vivo assay to effectively identify compounds that had inhibitory or leishmanicidal activity it was critical that the cultured splenocytes support the replication of the parasite in the absence of active drug over the course of the ex vivo culture. Additionally, since the luciferase-expressing episomal vector could be gradually lost after hamster inoculation, we had to establish the feasibility of quantifying parasite numbers in the ex vivo system. We found that the luciferase activity of amastigotes strongly correlated with the number of parasites counted by microscopy at the different times post-infection (R^2^ = 0.99, *p*<0.0001; Figure 2A). Luminometry clearly detected amastigote viability in the ex vivo system and most importantly demonstrated that the parasite numbers increased over 48 hrs of culture in the absence of test compound (*p<*0.05, at 48 h; Fig 2B), indicating ongoing replication of the parasite. This was confirmed by microscopic demonstration of an increase in the percentage of infected macrophages (Fig. 2C) and an increase in number of amastigotes per macrophage over the course of the culture (Fig. 2D and compare Fig. 2E to 2F). Because the increase in luminometric counts leveled off at 48 hrs of *ex vivo* culture the drug screening and EC_50_ determinations were completed at this time point. However, empiric testing of a subset of compounds in 72 and 96 hr splenic explant cultures showed that culturing the explants with and without the test compound for up to 96 hrs had no effect on the calculated EC_50_.

The 48 hr ex vivo culture also showed good discrimination between drug-treated and untreated control wells. The percent reduction in parasite burden in amphotericin B treated ex vivo splenic explants remained relatively constant over a range of 3,000–200,000 splenocytes per well (Table 2) indicating that there was considerable flexibility in the number of cells used for the screening assay. For the screening of chemical libraries 100,000 splenocytes per well were used. Thus, the number of splenocytes obtained from a single 21-day infected animal was sufficient to screen 13 plates containing a total of 1,040 compounds. The data in Table 2 also indicate that in situations where the number of animals were limited, or a very large number of compounds were going to be screened, the number of cells per well could be reduced substantially without compromising the discrimination between active and inactive compounds. We also found that variation of the ex vivo splenocyte number from 100,000 to 500,000 cells per well had no effect on the calculated EC_50_ of a test compound (data not shown).

**Table 2:** Determination of splenocyte numbers required for the ex vivo assay.

No. of cells	Parasite counts (photons/sec) a		Reduction in parasite burden b
(per well)	Untreated (control)	Treated (AMB)	(%)
3,125	0.7±0.0	0.1±0.1	83.5
6,250	1.2±0.2	0.2±0.0	86.3
12,500	2.8±0.9	0.4±0.3	86.2
25,000	5.3 ±0.8	0.7±0.0	86.4
50,000	14.2±0.4	2.5±0.5	82.3
100,000	51.0±10.1	8.5±0.2	83.3
200,000	145.2±0.4	34.8±3.7	76.0

a Parasite counts (mean ± SD) were determined by luminometry in splenocytes isolated from hamsters at 21 days p.i. and cultured *ex vivo* with amphotericin B (AMB), 0.126 µg/mL or DMSO control for 48 h.

b Reduction in parasite burden with reference to controls = 100 - [(parasite counts in treated splenocytes/parasite counts in control wells) x 100].

To confirm the quality of the assay in discriminating active from inactive compounds we calculated that the Z prime (Z') factor [31] in 3 different screening experiments using 36 different plates. The resulting value of 0.72±0.02 indicated that the assay could be considered as optimal (optimal assay, Z factor ≥0.5 but ≤1.0) [31]. To select the optimal drug concentration for the assays we compared the number of hits obtained after screening 80 different compounds at 10, 5 and 2.5 µM. Each of the concentrations gave similar numbers of hits (data not shown), so we chose the 10 µM concentration to be inclusive of as many hits as possible and limit the skewing of the hit selection toward toxic compounds.

### Validation and prioritization of screening hits

The initial screening using the *ex vivo* model showed that 239 of 4,035 compounds (5.9%) had a Z score of ≤–1.96 (equivalent to a *p*≤0.05) and could be qualified as screening ‘actives’ (Table 3). We did not observe microscopic evidence of cell death in the splenic explant cultures after 48 hrs of culture with the test compounds. Flow cytometry of propidium iodide stained splenocytes cultured with three representative test compounds (amphotericin B, tilorone, disulfuram) confirmed no loss of splenocyte viability over the 48 hr culture period (data not shown). To quantify cytotoxicity of a test compound we utilized an established cell line-based assay. We first excluded compounds that fell below an arbitrary cytotoxicity threshold in the HepG_2_ cell line (CC_50_< 10 µM), and after exclusion of 37 (1%) such (toxic) compounds, 202 (5%) hits were left for further validation studies (Table 3). For each of these hits, anti-leishmanial activity (EC_50_) was determined in the ex vivo splenic explant system. Comparison of these EC_50_ values with cytotoxicity (CC_50_) values in the HepG_2_ cell line allowed determination of an in vitro therapeutic index (IVTI: CC_50_/EC_50_). Based on a threshold IVTI score ≥5 [33], 84 compounds (2.1% of the total number of molecules screened) were identified as lead compounds (Tables 3, 4, S1). Of the 84 lead compounds, 15 (17%) had been shown previously to have anti-leishmanial activity (Table S2) and 69 (83%) had not been reported previously as having activity against *Leishmania* (Tables 4, S1). A substantial number of the latter, however, had been shown previously to have anti-infective activity against other classes of pathogens, while others were known as immune regulators, antidepressant, antipsychotics, or had no known function (Table 5).

**Table 3:** Hit and lead compounds identified from chemical libraries using the ex vivo splenic explant system.

		Initial Screening		Results after excluding toxic compounds^4^			
Compounds		Z score <−1.96 6		Hits 4		Leads (IVTI>5) 7	
Chemical Library	No.	No.	% 6	No.	% 6	No.	% 6
**NINDS Col II** 1	1,040	76	7.3	65	6.3	24	2.3
**NINDS NP** 2	800	51	6.4	46	5.8	12	1.5
**NCI** 3	2,195	112	5.1	91	4.1	48	2.2
**Total** 5	4,035	239	5.9	202	5	84	2.1

1 NINDS Col II (NINDS Collection II, MicroSource Discovery Systems).

2 NINDS NP (NINDS Natural Products Collection, MicroSource Discovery Systems).

3 Diversity set and Natural products set of the National Cancer Institute (NCI).

4 Compounds identified by Z score, excluding the toxic compounds (CC_50_ <10 µM for the HepG2 cell line).

5 Total number (No.) and % of compounds excluding amphotericin B, which is considered as the reference compound.

6 All percentages shown in the table refer to the total number of compounds of the initial screening. See Table S1 for compound details.

7 Leads identified using the in vitro therapeutic index (IVTI) calculated of the cell toxicity (CC_50_) and anti-Leishmania activity (EC_50_) ratio.

**Table 4:** Lead compounds newly identified or previously known to have anti-leishmanial activity.

			New		Known		Total	
Compound 1			No.	%	No.	%	No.	%
Heterocyclic compounds								
	1-ring							
		Furans	3	4	1	7	4	5
		Pyridines	1	1	0	0	1	1
		Piperidines	1	1	0	0	1	1
		Pyrans	0	0	3	20	3	4
			5	6	4	27	9	11
	2-ring							
		Isoquinolines	2	3	0	0	2	2
		Quinolines	12	17	0	0	12	14
		Purines	1	1	0	0	1	1
		Benzopyrans	2	3	0	0	2	2
		Bicyclocomp.	1	1	0	0	1	1
			18	26	0	0	18	21
	3-ring							
		Phenothiazines	5	7	1	7	6	7
		Acridines	1	1	0	0	1	1
		Xanthenes	3	4	0	0	3	4
		Phenanthridines	0	0	1	7	1	1
			9	13	2	13	11	13
Alkaloids			8	12	0	0	8	10
Hydrocarbons								
	Aromatics		8	12	1	7	9	11
	Terpenes		6	9	1	7	7	8
	Acyclic		1	1	0	0	1	1
			15	22	2	13	17	20
Polycyclic compounds								
	Macrocyclic		1	1	1	7	2	2
	Steroids		1	1	2	13	3	4
			2	3	3	20	5	6
Amines								
	Ethylamines		1	11	0	0	1	1
	Q. ammonium		2	9	0	0	2	2
	Polyamines		1	1	0	0	1	1
			4	6	0	0	4	5
Lactones			1	1	2	13	3	4
Onium comp.			2	3	1	7	3	4
Sulfur comp.			2	3	0	0	2	2
2 Others			3	4	1	7	4	5
Total			69	82	15	18	84	100

1 Chemical name according the MeSH Chemical Class Browser (Wolfram Demonstrations project), National Library of Medicine, National Institutes of Health, United States 2010.

2 Others: Amidines, Phenols, Carboxilic Acids, Organometallic compounds.

**Table 5:** Therapeutic category of anti-leishmanial compounds identified in the ex vivo splenic explant system.

*Anti-protozoa*	*Anti-bacterial*	*Anti-helmintic*
Elipticine	Nonactin	Pararosaniline Pamoate
Nigericin	Aklavine Hydrochloride	Naphthofuran d
Cepharanthine	Nigericin	Cetrimonium Bromide
Disulfiram	Naphthofuran d	Disulfiram
Tilorone	Streptovitacin A	
3-phenanthren a	5-methyl-8-quinolinol c	*Anti-fungal*
Benzalkonium Chloride	Securinine	Pararosaniline Pamoate
Physalin	Cepharanthine	Naphthofuran d
Chlorhexidine	Parthenicin	Cetrimonium Bromide
Cloxyquin	Disulfiram	Disulfiram
Tetrandrine	Salinomycin, Sodium	Acrisorcin
Clioquinol	Rubescensin A	Benzalkonium Chloride
Hexachlorophene	7-hydroxychlorpromazine	Clioquinol
Lasalocid Sodium	Lasalocid Sodium	Hexachlorophene
		Lasalocid Sodium
*Anti-neoplastic*	*Topical/Antiseptics*	Antimycin A
Aklavine Hydrochloride	5-methyl-8-quinolinol c	5-methyl-8-quinolinol c
Thaspine, Acetate	Cetrimonium bromide	
Ellipticine	Benzalkonium chloride	*Immune response regulator*
NSC 134754	Chlorhexidine	n6-Isopentenyladenine
Streptovitacin A	Clioquinol	Spermidine Trihydrochloride
Cepharanthine	Hexachlorophene	NSC 13480
Benzethonium Chloride	Cetylpyridinium Chloride	6,4′-Dimethoxyflavone
Rubescensin A		NSC 371488
10-Methyl-9-anthracenyl f	*Anti-viral*	Sumilit bbm
Tilorone	Nigericin	Securinine
Crassin	Cepharanthine	Cepharanthine
Tetrandrine	Trimethoxychalcone g	
NSC 305819	Tilorone	*Anti-obesity/Anti-psychotics*
NSC 371488	Benzalkonium Chloride	1h-benz[de]isoquinoline b
Clioquinol	Crassin Acetate	Nortriptyline Hydrochloride
Fastigilin B		Disulfiram
Thioxanthen-9 e	*Undefined* h	Orlistat
Crassin acetate		Thiomethylpromazine
6,4′-dimethoxyflavone		Chlorpromazine
		7-hydroxychlorpromazine
		Maprotiline hydrochloride

a 3-phenanthrenemethanol, alpha.-[(diethylamino)methyl]-,hydrochloride.

b 1h-benz[de]isoquinoline-1,3(2 h)- dione, 5-amino-2-[2-(diethylamino)ethyl]-

c 7-(2-(6-ethoxy-1-methyl-1lambda(5)-quinolin-2-yl)vinyl)-5-methyl-8-quinolinol.

d naphtho(2,1-b)furan, 4 methoxy-2 -nitro-

e thioxanthen-9-one, 4-(hydroxymethyl)-1-[(2-piperidinoethyl)amino.

f 10-Methyl-9-anthracenyl) methyl carbamimidothioic acid ester hydrochloride.

g 2′,4-dihydroxy-3,4′,6′ Trimethoxychalcone.

h Undefined activity: pyrimido[4,5-b]quinoline-2,4(3 h,10 h)-dione, 5-[[3-(dimethylamino)propyl]amino]- 3,10-dimethyl-,monohydrochloride]-; 17-(1-((2-(dimethylamino)ethyl)amino)ethyl) estra-1,3,5(10)-trien-3-ol; N1-(7-chloro-1,2,3,4-tetrahydro-9-acridinyl)-N3,N3-dimethyl-1,3-propanediamine; 2-[(2E)-2-hydroxyimino-4,6,6-trimethyl-1-cyclohex-3-enyl]-4,6,6-trimethyl-cyclohex-3-en-1-ol; 3-(12H-benzo[a]phenothiazin-12-yl)-N,N-dimethyl-1-propanamine; 5-Phenyl-1,2,3,5-tetrahydroimidazo[2,1-b]quinazolin-5-ol; 7-chloro-N-(4-(1-piperidinyl)cyclohexyl)-4-quinolinamine; 2-(3-dimethylaminopropylamino)-1,4-dihydroxy-anthracene-9,10-dione; 17-(Bis(2-hydroxyethyl)amino)androst- 5-en-3-ol; N1-7-chloro-4-quinolinyl)-N2-cyclohexyl-1,2-ethanediamine; 6-methoxy-N-[2-(2-piperidyl)ethyl]quinolin-8-amine.

In general, heterocyclic compounds were most highly represented among the lead compounds, comprising 55% of the total (Table 4). Eleven percent of the lead compounds were single-ring heterocyclic structures, 21% had 2-ring structures, 14% were classified as quinolines, and 7% were 3-ring phenothiazines. Ten percent of the leads were alkaloids and 20% were hydrocarbon structures composed of aromatics and terpenes (Tables 4, S1). The chemical libraries screened included large numbers of known bioactives and drugs so it was not surprising that 27 of 84 (32%) leads had been previously used clinically. Eleven of the lead compounds are recommended for topical use only (Table 5).

Three known anti-leishmanial drugs, fluconazole, pentamidine and miltefosine included in the libraries surprisingly did not show a significant Z score and were not identified as hit compounds in the screening. To understand the reason behind this finding, we determined the EC_50_ of these and other anti-leishmania drugs and lead compounds in both the ex vivo splenic explant and in vitro macrophage infection models (Table 6). Repeated testing of Miltefosine from the NCI chemical library found it to be inactive, but testing of freshly solubilized compound from a different commercial source was found to be highly active (EC_50_ = 1 µM). Thus it would appear that the miltefosine in the NCI library had degraded to an inactive form. Similarly, the EC_50_ calculated for the amphotericin B present in the library (in DMSO vehicle) was 10.7±0.9 µM, whereas freshly solubilized amphotericin B deoxycholate (Sigma) had an EC_50_ of 0.24±0.02 µM. In the case of pentamidine, the Z score of −1.87 was just outside the threshold for statistical significance (a Z score of −1.96 is equivalent to p = 0.05) and determination of the EC_50_ for pentamidine revealed that it was active in the ex vivo splenocytes system (EC_50_ = 3 µM). Fluconazole had no activity detected by either the screen or determination of the EC_50_. Collectively these data indicate that the ex vivo system is a robust approach to identification of new compounds, but that like any screen, it is only as good as the quality of the compounds (libraries) screened.

**Table 6:** Comparative anti-*Leishmania donovani* activity of lead compounds in the ex vivo splenic explant model and in vitro infected peritoneal macrophages.

	*Ex vivo* model	Peritoneal Macrophages
Compound	EC_50_ (µM; mean±SE) 1	EC_50_ (µM; mean±SE) 1
Amphotericin B	0.09±0.03	0.11±0.01
Miltefosine	1.02±0.08	2.28±0.21
Pentamidine	3.02±0.34	0.89±0.37
Fluconazole	>250	>250
Meglumine antimoniate	269.60±57.50	146.30±42.01
Antimycin A	1.27±1.07	1.67±1.05
Disulfiram	0.31±0.03	0.16±0.03
Monensin A	0.85±0.68	0.23±0.15
Nortriptyline	6.03±0.28	2.65±0.35
Tilorone	0.84±0.47	1.48±0.35

1 Effective concentration of the compound that killed 50% of the parasites (EC_50_). Determined at 48 h of culture by luminometry.

### Comparison of compounds tested in the ex vivo explant and in vitro macrophage infection models

To further validate the ex vivo splenic explant model for drug screening we determined the EC_50_ of a subset of 10 compounds (including 5 known anti-leishmania drugs) using both the ex vivo and in vitro infected macrophage systems. Infected splenocytes and in vitro infected macrophages were cultured under the same conditions for 48 hrs in the presence or absence of serial dilutions of test compound. We found good correlation between the two systems (R^2^ = 0.78; *p* = 0.027).

## Discussion

New drugs are desperately needed for the treatment of VL, and innovative approaches are needed to identify new lead compounds and classes of compounds that can enter the pipeline of lead optimization and therapeutic testing. We describe here a novel approach to drug discovery for VL. We sought to develop a model system through which the activity of test compounds could be determined within the physiological and immunological environment of the site of infection. Since the host immune response is known to have profound influence on the treatment and outcome of *Leishmania* infection, and VL is characterized by suppression of cellular immune function, we felt it was critical that new compounds be screened for activity within the immunopathological milieu found at the site of the host-parasite interaction during active disease. The ex vivo splenic explant culture used for the drug screening reported here was established from *L. donovani* infected hamsters that demonstrated the immunopathological features of active VL in that 1) there was enlargement of the spleen, 2) the splenic parasite burden was rapidly increasing, 3) there was loss of antigen-specific T cell reactivity [34], 4) the splenic T cell population was contracting while the B cell and macrophage populations were expanding [35], and 5) the splenic macrophages had acquired an alternatively activated phenotype. Furthermore, the cultured spleen cell explants contained the full repertoire of spleen cells and supported ongoing parasite replication during the 48 hrs of exposure to test compound.

The novel ex vivo splenic explant model showed excellent discrimination between active and inactive compounds in a medium-throughput screening format. Known anti-*Leishmania* drugs were readily identified upon the screening of the chemical libraries, confirming the ability of the ex vivo model to identify active compounds. Furthermore, the practical advantages of the ex vivo approach presented here are numerous. The model uses a *Leishmania* strain that is episomally transfected with the firefly luciferase reporter gene, which has been successfully used by others to quantify in vitro and in vivo infections [19], [36]. The light emission of luciferase-tranfected parasites allows one to quantify the amastigote numbers in the samples by extrapolation to a standard amastigote curve, making this approach readily adaptable to a quantitative high throughput assay. The relative high cost of the luciferin substrate is offset by the radical decrease in labor, high sensitivity, and reproducibility of the method. Since in the *ex vivo* model the cells are harvested from infected animals and the screening is carried out in 96-well plates, no parasitological expertise to quantify amastigotes is necessary, and the time consuming and potentially biased microscopy-based evaluations that preclude automation are avoided. The fact that the luciferase-transfected *L. donovani* are selected by their resistance to the aminoglycoside G418 (Geneticin) suggests that related compounds would not be identified in the screen, however we found that another aminoglycoside, neomycin, was identified as a compound with activity against the transgenic *L. donovani* used in the ex vivo system. Other approaches that have used GFP-transfected *Leishmania* showed that the sensitivity of measuring fluorescence is not sufficient to enable microplate screening, and consequently a more demanding analysis using a flow cytometer is required [37]. The requirement of animals to establish the ex vivo splenic explant model is minimal because the cells obtained from a single infected hamster are sufficient to screen more than 1,000 compounds.

Although axenically cultured amastigotes have been used to screen drug candidates on a small scale [38], [39] not all parasite strains can be cultured as axenic amastigotes. It has been shown that *in vitro* assays that utilize intracellular amastigotes in macrophage cell lines correlate better with the response to treatment in vivo compared with assays in which promastigotes are used [40]. Screenings that involve in vitro infected macrophages have the technical limitation of difficulty in removal of extracellular promastigotes, and the theoretical concern that the non-macrophage immune regulatory cells are absent. Our results showed that the anti-*Leishmania* activity of lead compounds in the ex vivo spleen cell explants differed substantially from that found in cultured promastigotes, and to a lesser extent from freshly isolated tissue amastigotes. Thus, some of the lead compounds would not have been identified if promastigotes or cell-free amastigotes had been used. The lack of correlation between these techniques suggests that this ex vivo screening system can be exploited for the discovery of drugs targeting metabolic pathways of amastigotes in the host cells and/or interacting with the immune system. Accordingly, review of the published literature about the mechanisms of action of the 84 lead compounds revealed that the likely mode of action or parasite targets were the cell membrane (21%), cell metabolism (17%), the host immune response (13%), apoptosis (7%), and DNA interaction (6%).

The screening of compounds for anti-Leishmania activity in the *ex vivo* model, coupled with screening for toxicity using the HepG_2_ hepatocyte cell line, enabled the selection of 84 lead compounds, 69 of which had not been identified previously to have anti-*Leishmania* activity. While the use of cell lines to predict in vivo toxicity has some limitations [41], the HepG_2_ cell line is considered a good predictor of human toxicity [42], [43]. Although measurement of ATP in viable cells this method is one of the most reliable methods to estimate cell toxicity [44] multiparametric toxicity testing (based on features such as apoptosis markers and membrane integrity) and other in vitro models may be desirable to help in the assessment of hepatic cytotoxicity [45] and further lead optimization [44]. Ultimately, the final selection of lead compounds will require in vivo studies to identify dose limiting toxicities and to evaluate whether the compound's pharmacokinetic and ADME (absorption/distribution/metabolism/excretion) properties make it suitable for use in VL. Already it is clear that some of the lead compounds (e.g. topical and antiseptic agents) are unlikely to be useful in treating systemic infection.

Compounds containing the heterocyclic quinoline ring system were frequently identified in the screen as active inhibitors, representing 14% of the lead compounds. The quinoline leads could be further divided into several distinct classes. A number of the leads bear obvious structural relation to antimalarial quinolines, including several 4-aminoquinolines with basic side chains, as well as 8-aminoquinolines and a 2-arylquinoline derivative containing the core structure of the antimalarial drug mefloquine. Also represented were 8-hydroxyquinoline antifungals like clioquinol as well as more novel dimeric 2-aminoquinolines and quinoline diones. Several of the quinoline-containing compounds showed activity at micromolar concentrations and low toxicity to the HepG_2_ cell line, suggesting that they are good leads for optimization. Previous reports have described other quinoline-containing compounds with anti-*Leishmania* activity. For example, a 2-substitued quinoline alkaloid reduced by 79.6% the parasite load in the liver of BALB/c mice infected with *L. donovani* [46]. Sitamaquine, an 8-aminoquinoline, completed a phase II study for treatment of VL [47], and more recently, DNDi has incorporated synthetic 2-quinoline derivatives as part of the strategy to develop new anti-*Leishmania* drugs (http://www.dndi.org/newsletters/n18/edito.php). The quinoline ring system is common in known drugs and other bioactive molecules and such compounds can exhibit distinct bioactivities depending on their specific structures. Previously, quinoline derivatives have been reported to affect electron transport and generate lethal oxidative radicals against *Leishmania*, and also to inhibit cysteine proteases [48], a gene family important for *Leishmania* virulence [49]. Other quinoline derivatives have also been shown to alter the vesicle trafficking and endocytosis in *Plamodium falciparum* [50]. Our results, together with these prior observations, suggest the likely possibility that multiple distinct bioactivities are represented among the quinoline leads identified here and that multiple pharmacologically orthogonal candidates might be selected for further lead optimization studies.

Alkaloids also represented an important fraction of the lead compounds (10%) identified in the ex vivo screening. This parallels the observation that alkaloids derived from natural products have been found to be active against *Leishmania* species (reviewed by [51]).

Disruption or alteration of membrane function was identified as a mode of action for a number of compounds (ionophores, quaternary ammonium salts and tricyclic anti-depressants [52]) that were identified as leads in our screen (Tables 4, S1). In addition to a possible direct effect on the parasite membrane these inhibitors have the potential to affect the membrane integrity of the parasitophorous vacuole in which *Leishmania* resides, and perturb the capability to regulate the intraphagosomal trafficking of essential substrates for parasite survival [53]. Specific inhibition of biosynthesis of phosphatidylcholine has been proposed for some quaternary ammonium salts active against *L. major* promastigotes and *L. braziliensis* [54]. One of the leads identified here, cetrimonium bromide, is a cationic surfactant that closely resembles the structure of hexadecylphosphocholine (miltefosine), a drug in current use for VL. It has also been shown to inhibit choline kinase that regulates the biosynthesis of the most abundant phospholipid (phosphatidylcholine) in *Plasmodium falciparum* [55]. Because these lead compounds are recommended only for topical and not systemic administration they will not be drug candidates for VL, but identification of their molecular targets could facilitate new screening campaigns that could identify lead compounds more suitable for systemic use.

Phenothiazine compounds related to the tricyclic antidepressants constituted 7% of the leads, but their favorable IVTI was primarily a result of their low toxicity, rather than their good anti-*Leishmania* activity (EC_50_ 11±2.7 µM). This class of drugs was identified as having in vitro anti-*Leishmania* activity 30 years ago, but the absence in the literature of any in vivo therapeutic data would suggest that may not have good clinical efficacy. Other phenothiazine compounds are potent inhibitors of parasite trypanothione reductase, a key enzyme involved in many redox defenses of *Leishmania*. However, no correlation between anti-leishmanial efficacy and the potency of several trypanothione reductase inhibitors was found [56].

A broad range of biological effects have been recognized for the hydrocarbon terpenes, which represented 8% of the lead compounds. After alkaloids, natural product triterpenes are the inhibitors found most frequently as having activity against *Leishmania* spp. [51]. The terpenoids of the plant family *Asteraceae parthenolide* have been shown to inhibit amastigotes and promastigotes of *L. amazonensis* [57]. An analog of Harmine, a beta-carboline amine alkaloid identified in our screening, reduced the spleen parasite load by 40–80% in hamsters through necrosis and non-specific parasite membrane damage [58]. However, the selection of inhibitors targeting parasite specific metabolic pathways without altering the host's cell would be more desirable.

In summary, the ex vivo splenic explant model, which is comprised of the full repertoire of host cells including chronically infected macrophages and fibroblasts, enabled the identification of small molecules that have anti-*Leishmania* activity within the immunopathological milieu that closely resembles the in vivo features of progressive VL. The inclusion of the complex biological interactions between the parasite and host within the test system may also favor the identification of lead compounds that act at multiple targets [59]. The standardized approach presented here identified a number of compounds that had good potency, including some that are currently in clinical use for other indications. Further study in animal infection models seems a prudent next step for those agents already used systemically. Among the other lead compounds identified in this work are several interesting chemotypes (e.g. quinolines) that are good candidates for a lead optimization. The identification of highly active anti-leishmanial compounds in this ex vivo model of VL could contribute greatly to new drug discovery for this serious and neglected disease.

## Supporting Information

Table S1 Lead compounds with anti-*Leishmania donovani* activity identified in the screening using the ex vivo splenic explant model.
(0.21 MB DOC)

Table S2 Lead compounds known to have anti-*Leishmania* activity identified by screening in the ex vivo splenic explant model.
(0.06 MB DOC)
