# Supplementary material for: Identification of Small Molecule Lead Compounds for Visceral Leishmaniasis Using a Novel Ex Vivo Splenic Explant Model System
Source: PLoS Negl Trop Dis. 2011 Feb 15;5(2):e962. doi: 10.1371/journal.pntd.0000962 (PMC3039689; doi:10.1371/journal.pntd.0000962)
Supplement: Table S1 — Lead compounds with anti-Leishmania donovani activity identified in the screening using the ex vivo splenic explant model. (0.21 MB DOC) [file pntd.0000962.s001.doc]

Table S1. Lead compounds with anti-<i>Leishmania donovani<i> activity identified in the screening using the <i>ex vivo<i> splenic explant model

| **CHEMICAL NAME ^5^** | | | |  |  | **HepG_2_** | **Ex vivo model** | | **IVTI ^2^** |
| --- | --- | --- | --- | --- | --- | --- | --- | --- | --- |
|  |  |  |  |  |  | **CC_50_ (µM)** | **EC_50_ (µM) ^1^** | | **ratio** |
|  | | | | **CAS or NSC** | **Activity ^4^** | **Mean** | **Mean** | **SE ^3^** |  |
| **HYDROCARBONS** | | | |  |  |  |  |  |  |
|  | *HYDROCARBONS CYCLIC AND POLYCYCLIC* | | |  |  |  |  |  |  |
|  |  | AROMATICS | |  |  |  |  |  |  |
|  |  |  | Hexachlorophene | CAS 70-30-4 | u | 71.4 | 13.6 | 2.0 | 5.3 |
|  |  |  | 3-(9-tert-butyl-3-azaspiro[5.5]undec-3-yl)-N,N-dimethyl-1-propanamine | NSC 64875 | u | 61.8 | 6.1 | 2.4 | 10.1 |
|  |  |  | Maprotiline Hydrochloride | CAS 10347-81-6 | u | 57.9 | 10.8 | 0.8 | 5.3 |
|  |  |  | Aklavine Hydrochloride | CAS 60504-57-6 | u | 14.0 | 0.7 | 0.5 | 20.3 |
|  |  |  | 2-((3-(dimethylamino)propyl)amino)-1,4-dihydroxyanthra-9,10-quinone | CAS 3900-43-4 | u | 75.1 | 6.7 | 2.2 | 11.3 |
|  |  |  | 3-Phenanthrenemethanol, .alpha.-[ (diethylamino)methyl]-, hydrochloride | CAS 5341-13-9 | u | 54.2 | 8.9 | 0.9 | 6.1 |
|  |  |  | Tilorone | 27591-69-1 | u | 46.8 | 7.7 | 1.0 | 6.1 |
|  |  |  | Pararosaniline Pamoate | CAS 7232-51-1 | u | 37.1 | 2.7 | 1.2 | 13.9 |
|  |  |  | Sertraline Hydrochloride | 79559-97-0 | k | 82.5 | <20-11.4 | | 4.1 |
|  | *HYDROCARBONS, TERPENES* | | |  |  |  |  |  |  |
|  |  |  | 17beta-[Bis(2-hydroxyethyl)amino]androst-5-en-3beta-ol | CAS 6956-93-0 | u | 88.5 | 7.5 | 2.3 | 11.8 |
|  |  |  | Rubescensin A | CAS 28957-04-2 | u | 69.1 | 11.0 | 1.3 | 6.3 |
|  |  |  | Crassin | NSC 210236 | u | 51.6 | 8.9 | 2.9 | 5.8 |
|  |  |  | Crassin Acetate | CAS 28028-68-4 | u | 41.1 | 7.7 | 0.1 | 5.3 |
|  |  |  | Fastigilin B | NSC 176503 | u | 44.8 | 8.9 | 1.3 | 5.1 |
|  |  |  | Parthenicin | CAS 508-59-8 | u | 64.5 | 8.7 | 0.2 | 7.4 |
|  |  |  | Parthenolide | CAS 20554-84-1 | k | 81.1 | 13.3 | 1.6 | 6.1 |
|  | *HYDROCARBONS, ACYCLIC* | | |  |  |  |  |  |  |
|  |  |  | Sumilit BBM | CAS 85-60-9 | u | 71.7 | 9.4 | 5.3 | 7.6 |
| **AMINES** | | | |  |  |  |  |  |  |
|  |  | ETHYLAMINES | |  |  |  |  |  |  |
|  |  |  | Dehydroabietylamine | NSC 65238 | u | 32.9 | 5.8 | 2.9 | 5.6 |
|  |  | QUATERNARY AMMONIUM COMPOUNDS | |  |  |  |  |  |  |
|  |  |  | Cristox Oxime | CAS 7248-80-8 | u | 140.5 | 10.9 | 1.7 | 12.9 |
|  |  |  | Cetrimonium Bromide | CAS 57-09-0 | u | 42.9 | 6.1 | 0.4 | 7.0 |
|  |  | POLYAMINES | |  |  |  |  |  |  |
|  |  |  | Spermidine Trihydrochloride | ID 01503940 | u | 132.4 | 20.0 | 0.0 | 6.6 |
| **AMIDINES** | | | |  |  |  |  |  |  |
|  |  | GUANIDINES | |  |  |  |  |  |  |
|  |  |  | Chlorhexidine | CAS 3697-42-5 | u | 60.5 | 10.3 | 0.2 | 5.9 |
| **LACTONES** | | | |  |  |  |  |  |  |
|  |  | MACROLIDES | |  |  |  |  |  |  |
|  |  |  | Orlistat | CAS 96829-58-2 | u | 161.3 | 24.9 | 14.1 | 6.5 |
|  |  | Amphotericin B (included in the library) | | CAS-1397-89-3 | k | 104.5 | 10.7 | 0.9 | 9.8 |
|  |  | ANTIMYCIN A | | CAS 1397-94-0 | k | 58.4 | 11.6 | 4.8 | 5.1 |
| **SULFUR COMPOUNDS** | | | |  |  |  |  |  |  |
|  |  | THIOUREA | |  |  |  |  |  |  |
|  |  |  | (10-methyl-9-anthracenyl) carbamimidothioic acid methyl ester hydrochloride | CAS 59474-01-0 | u | 23.7 | 3.5 | 0.5 | 6.7 |
|  |  |  | Diethylaminomethoxymethanedithioic acid | NSC 24076 | u | 83.7 | 3.7 | 2.9 | 22.9 |
| **ONIUM COMPOUNDS** | | | |  |  |  |  |  |  |
|  |  | QUATERNARY AMMONIUM COMPOUNDS | |  |  |  |  |  |  |
|  |  |  | Benzethonium Chloride | CAS 121-54-0 | u | 57.6 | 8.6 | 1.3 | 6.7 |
|  |  |  | Benzalkonium Chloride | CAS 8001-54-5 | u | 38.6 | 6.4 | 2.6 | 6.1 |
|  |  |  | Methylbenzethonium Chloride | CAS 1320-44-1 | k | 53.2 | 7.1 | 0.1 | 7.5 |
| **PHENOLS** | | | |  |  |  |  |  |  |
|  |  |  | Chlorocresol | CAS 59-50-7 | k | 138.2 | 8.0 | 1.2 | 17.2 |
| **CARBOXYLIC ACIDS** | | | |  |  |  |  |  |  |
|  |  | CARBAMATES | |  |  |  |  |  |  |
|  |  |  | Disulfiram | CAS 97-77-8 | u | 53.0 | 8.1 | 0.9 | 6.6 |
| **ORGANOMETALLIC COMPOUNDS** | | | |  |  |  |  |  |  |
|  |  | ARSENICALS | |  |  |  |  |  |  |
|  |  |  | 4-[(4-arsonophenyl)methyl]phenyl]arsonic acid | NSC 48300 | u | 34.5 | 6.9 | 2.3 | 5.0 |
| **HETEOCYCLIC COMPOUNDS** | | | |  |  |  |  |  |  |
|  | *ALKALOIDS* | | |  |  |  |  |  |  |
|  |  |  | Thaspine Acetate | CAS 74578-01-1 | u | 51.4 | 2.7 | 1.2 | 19.3 |
|  |  |  | Virosecurinine | CAS 6704-68-3 | u | 120.9 | 8.8 | 0.5 | 13.8 |
|  |  |  | Cepharanthine | CAS 481-49-2 | u | 86.3 | 11.4 | 0.9 | 7.5 |
|  |  |  | Securinine | CAS 5610-40-2 | u | 47.7 | 6.2 | 0.1 | 7.7 |
|  |  |  | Harmol Hydrochloride | CAS 40580-83-4 | u | 60.2 | 11.2 | 2.5 | 5.4 |
|  |  | BENZYLISOQUINOLINES | |  |  |  |  |  |  |
|  |  |  | Tetrandrine | CAS 518-34-3 | u | 31.4 | 5.6 | 0.9 | 5.6 |
|  |  | INDOLE ALKALOIDS | |  |  |  |  |  |  |
|  |  |  | Ellipticine | CAS 519-23-3 | u | 30.7 | 2.4 | 0.8 | 12.7 |
|  |  |  | 2-[1-[(2-chlorophenyl)methyl]-2-methyl-5-methylsulfanyl-indol-3-yl]ethanamine | NSC 17383 | u | 71.8 | 9.4 | 5.3 | 7.7 |
|  | *HETEROCYCLIC COMPOUNDS, 1-RING* | | |  |  |  |  |  |  |
|  |  | FURANS | |  |  |  |  |  |  |
|  |  |  | Lasalocid Sodium | CAS 25999-31-9 | u | 45.8 | 8.9 | 0.3 | 5.2 |
|  |  |  | Naphtho(2,1-b)furan, 4-methoxy-2-nitro- | CAS 75965-72-9 | u | 64.3 | 6.8 | 2.3 | 9.5 |
|  |  |  | 2-[4-(5-phenylfuran-2-yl)phenyl]-4,5-dihydro-1H-imidazole chloride | NSC 17600 | u | 42.9 | 7.5 | 2.5 | 5.7 |
|  |  |  | Monensin Sodium | CAS 22373-78-0 | k | 72.9 | 2.4 | 2.0 | 29.9 |
|  |  | PYRIDINES | |  |  |  |  |  |  |
|  |  |  | Cetylpyridinium Chloride Monohydrate | CAS 6004-24-6 | u | 37.6 | 1.5 | 0.6 | 25.9 |
|  |  | PIPERIDINE | |  |  |  |  |  |  |
|  |  |  | Streptovitacin A | CAS 523-86-4 | u | 32.4 | 3.7 | 0.3 | 8.8 |
|  |  | PYRANS | |  |  |  |  |  |  |
|  |  |  | Salinomycin, Sodium | CAS 53003-10-4 | k | 48.4 | 7.4 | 1.4 | 6.5 |
|  |  |  | Nigericin, monosodium salt | CAS 28643-80-3 | k | 34.3 | 3.1 | 2.3 | 10.9 |
|  |  |  | Narasin | CAS 55134-13-9 | k | 73.0 | 13.3 | 1.3 | 5.5 |
|  | *HETEROCYCLIC COMPOUNDS, 2-RING* | | |  |  |  |  |  |  |
|  |  | ISOQUINOLINES | |  |  |  |  |  |  |
|  |  |  | 3-ethyl-9,10-dimethoxy-2-(1,2,3,4-tetrahydro-isoquinolin-1-ylmethyl)-1,6,7,11b-tetrahydro-4H-pyrido(2,1-a)isoquinoline | NSC 134754 | u | 24.0 | 2.7 | 1.3 | 8.9 |
|  |  |  | 2-​[(6,7-​dimethoxy-​1,2,3,4-​tetrahydroisoquinolin-​1-​yl)methyl]-​3-​ ethyl-​4,6,7,11b-​tetrahydro-​1H-​pyrido[2,1-​a]isoquinoline | NSC 134755 | u | 76.2 | 8.8 | 2.2 | 8.7 |
|  |  | QUINOLINES | |  |  |  |  |  |  |
|  |  |  | 6-methoxy-N-(2-(2-piperidinyl)ethyl)-8-quinolinamine | CAS 5429-25-4 | u | 107.5 | 5.1 | 1.1 | 21.1 |
|  |  |  | N1-(7-chloro-4-quinolinyl)-N2-cyclohexyl-1,2-ethanediamine | CAS 5427-42-9 | u | 46.2 | 3.9 | 2.3 | 11.9 |
|  |  |  | 7-chloro-N-(4-(1-piperidinyl)cyclohexyl)-4-quinolinamine | CAS 6632-99-1 | u | 67.4 | 6.3 | 1.1 | 10.6 |
|  |  |  | 1H-Benz[de]isoquinoline-1,3(2H)-dione, 5-amino-2-[2-(diethylamino)ethyl]- | CAS 69408-82-8 | u | 69.3 | 8.4 | 2.2 | 8.2 |
|  |  |  | 7-(2-(6-Ethoxy-1-methyl-1.lambda.~5~-quinolin-2-yl)vinyl)-5-methyl-8-quinolinol; Quinolinium, {6-ethoxy-2-[2-(8-hydroxy-5-methyl-7-quinolyl)vinyl]-1-methyl-} iodide | CAS 13953-25-8 | u | 46.0 | 5.7 | 0.9 | 8.1 |
|  |  |  | Pyrimido[4,5-b]quinoline-2,4(3H,10H)-dione, 5-[[3-(dimethylamino)propyl]amino]-3,10-dimethyl-, monohydrochloride | NSC 373989 | u | 49.5 | 6.5 | 1.3 | 7.6 |
|  |  |  | 4-methyl-6-((4-methyl-2-(1-piperidinyl)-6-quinolinyl)methyl)-2-(1-piperidinyl)quinoline | CAS 5463-36-5 | u | 109.2 | 18.4 | 5.3 | 5.9 |
|  |  |  | Cloxyquin | CAS 130-16-5 | u | 94.8 | <20-12.8 | | 5.7 |
|  |  |  | 2-phenylbenzo[h]quinolin-4-yl)(2-piperidinyl)me | NSC 13480 | u | 28.7 | 5.0 | 1.0 | 5.7 |
|  |  |  | 2-(4-chlorophenyl)quinolin-4-yl]-(2-piperidyl)methanol | CAS 5428-80-8 | u | 38.4 | 6.7 | 2.0 | 5.7 |
|  |  |  | 1-(2,8-bis(trifluoromethyl)-4-quinolinyl)-3-(tert-butylamino)-1-propanol | NSC 305819 | u | 44.2 | 7.9 | 1.0 | 5.6 |
|  |  |  | Clioquinol | CAS 130-26-7 | u | 60.0 | 11.3 | 0.3 | 5.3 |
|  |  | PURINES | |  |  |  |  |  |  |
|  |  |  | N6-Isopentenyladenine | CAS 2365-40-4 | u | 225.0 | 20.0 | 0.0 | 11.2 |
|  |  | BENZOPYRANS | |  |  |  |  |  |  |
|  |  |  | 2',4-dihydroxy-3,4',6'-trimethoxychalcone | CAS 112572-59-5 | u | 105.7 | 14.6 | 3.2 | 7.2 |
|  |  |  | 6,4'-dimethoxyflavone | CAS 54401-47-7 | u | 95.0 | 17.9 | 2.1 | 5.3 |
|  |  | BICYCLO COMPOUNDS | |  |  |  |  |  |  |
|  |  |  | 5-phenyl-3,10-dihydro-2H-imidazo[2,1-b]quinazolin-5-ol | CAS 68210-55-9 | u | 106.8 | 12.0 | 1.5 | 8.9 |
|  | *HETEROCYCLIC COMPOUNDS, 3 RING* | | |  |  |  |  |  |  |
|  |  | PHENOTHIAZINES | |  |  |  |  |  |  |
|  |  |  | Nortriptyline Hydrochloride | CAS 894-71-3 | u | 52.6 | 7.8 | 1.5 | 6.7 |
|  |  |  | Thiomethylpromazine | CAS 1039-59-4 | u | 83.9 | 13.0 | 3.5 | 6.5 |
|  |  |  | Mequitazine,10-(3-Quinuclidinylmethyl)phenothiazine | CAS 29216-28-2 | u | 50.3 | 8.5 | 1.5 | 5.9 |
|  |  |  | 3-(12H-benzo[a]phenothiazin-12-yl)-N,N-dimethyl-1-propanamine | CAS 5453-77-0 | u | 66.6 | 11.5 | 4.9 | 5.8 |
|  |  |  | 7-Hydroxychlorpromazine | CAS 2095-62-7 | u | 60.1 | 11.0 | 2.0 | 5.4 |
|  |  |  | Chlorpromazine | CAS 50-53-3 | k | 96.1 | >20-10.3 | | 6.3 |
|  |  | ACRIDINES | |  |  |  |  |  |  |
|  |  |  | Acrisorcin | CAS 7527-91-5 | u | 50.9 | 8.0 | 0.3 | 6.4 |
|  |  | XANTHENES | |  |  |  |  |  |  |
|  |  |  | Thioxanthen-9-one, 4-(hydroxymethyl)-1-[(2-piperidinoethyl)amino]-; 9H-Thioxanthen-9-one, 4-(hydroxymethyl)-1-[[2-(1-piperidinyl)ethyl]amino]- | CAS 16140-27-5 | u | 56.0 | 9.1 | 5.5 | 6.1 |
|  |  |  | 9-(3,4-Dichlorophenyl)-2,7-bis(dimethylaminomethyl)-3,4,5,6,7,9-hexahydro-2H-xanthene-1,8-dione | NSC 371488 | u | 49.8 | 9.4 | 2.9 | 5.3 |
|  |  |  | N-(7-chloro-1,2,3,4-tetrahydroacridin-9-yl)-N',N'-dimethyl-propane-1,3-diamine | CAS 6297-84-3 | u | 58.8 | 6.4 | 2.4 | 9.2 |
|  |  | PHENANTHRIDINES | |  |  |  |  |  |  |
|  |  |  | Homidium Bromide | CAS 1239-45-8 | k | 58.8 | 11.9 | 1.4 | 8.5 |
|  | *POLYCYCLIC COMPOUNDS* | | |  |  |  |  |  |  |
|  |  | MACROCYCLIC COMPOUNDS | |  |  |  |  |  |  |
|  |  |  | Valinomycin | CAS 2001-95-8 | k | 10.7 | 0.02 | 0.0 | 537.4 |
|  |  |  | Nonactin | CAS 6833-84-7 | u | 23.7 | 0.1 | 0.0 | 198.9 |
|  |  | STEROIDS | |  |  |  |  |  |  |
|  |  |  | 17-(1-((2-(dimethylamino)ethyl)amino)ethyl)estra-1,3,5(10)-trien-3-ol | CAS 72-37-7 | u | 51.6 | 6.2 | 2.2 | 8.4 |
|  |  |  | Physalin B | NSC 287088 | k | 50.7 | 8.6 | 1.2 | 5.9 |
|  |  |  | Digitonin | CAS 11024-24-1 | k | 42.4 | 8.2 | 0.3 | 5.2 |

<sup>1<sup> EC<sub>50<sub>: Effective concentration of drug that kills 50% of the parasites. Determined at least in two different experiments

<sup>2<sup> IVTI : In vitro therapeutic index is the ratio between the cell toxicity (concentration of drug that kills 50% of cells; CC<sub>50<sub>) to the HepG<sub>2<sub>cell line and the anti-Leishmania efficacy (EC<sub>50<sub>) of the compound. IVTI Determinations in compounds with > symbol were estimated using the highest dilution tested.

<sup>3<sup> SE : Standard error of the mean obtained in two or three different experiments

<sup>4<sup> Activity : k= known inhibitor of <i>Leishmania<i> spp.; u=not reported as <i>Leishmania<i> spp. inhibitor (PubMed data Base, 2010).

<sup>5<sup> Chemical name according the MeSH Chemical Class Browser (Wolfram Demostrations project); National Library of Medicine, National Institutes of Health, United States.
