# Supplementary material for: Identification of Small Molecule Lead Compounds for Visceral Leishmaniasis Using a Novel Ex Vivo Splenic Explant Model System
Source: PLoS Negl Trop Dis. 2011 Feb 15;5(2):e962. doi: 10.1371/journal.pntd.0000962 (PMC3039689; doi:10.1371/journal.pntd.0000962)
Supplement: Table S2 — Lead compounds known to have anti-Leishmania activity identified by screening in the ex vivo splenic explant model. (0.06 MB DOC) [file pntd.0000962.s002.doc]

Table S2. Lead compounds known to have anti-<i>Leishmania<i> activity identified by screening in the <i>ex vivo<i> splenic explant model.

|  | EC_50_ (μM) ^a^ | | Compounds in Clinical Use | |
| --- | --- | --- | --- | --- |
| Compound | Mean | SE |  | Leishmania spp. Activity ^b^ |
| Chlorocresol | 8.02 | 1.17 | Antiseptic and preservative | L. d. ^1^; L. p. ^2^ |
| Neomycin sulfate | 6.72 | 6.64 | Bacterial infections | L. m. ^4^ |
| Amphotericin B | 10.7 | 0.9 | VL^5^ , ACL ^6^ | L. d.; L. m. ^4^, L.i. ^3^ |
| Methylbenzethonium chloride | 7.06 | 0.07 | CL in combination with Paromomycin ^7^ | L. m. ^7^, L. b. ^7^, L. p. ^8^, L. m. ^9^ |
| Chlorpromazine | 15.1 | 4.8 | Antipsychotic ^10^ | L. m. L. ae., L. m. ^11^ , L. d.^c 12^ |
| Sertraline hydrochloride | 15.7 | 4.3 | Antidepressant ^13^, sexual disorders ^14,15^ | L. d. ^c^ ^16^ |
|  | | | Compounds Tested in Experimental models | |
| Valinomycin | 0.02 | 0.00 | Membrane potential studies ^17^ | L. d. ^20^, L. m.^c 18^ |
| Monensin sodium | 2.44 | 1.97 | Ionophore antibiotic used in animals ^22^ and biochemical studies ^19^ | L. d.^20^ |
| Homidium bromide | 11.89 | 1.43 | Molecular biology techniques | L. t.^21^ |
| Salinomycin, sodium | 7.44 | 1.40 | Ionophore antibiotic used in animals ^22^ | L. d. ^23^ |
| Parthenolide | 13.32 | 1.60 | NF-kappa B inhibitor ^24^ | L.a.^c 25^ |
| Physalin B | 8.59 | 1.24 | Antiinflamatory action ^26^ | L. a.^c^ ^27^ |
| Narasin | 13.28 | 1.27 | Treatment of enteritis in broiler chickens ^28^ | L. d.^23^ |
| Antimycin A | 11.55 | 4.75 | Inhibitor of mitrochondrial respiration ^29^ | L. a. ^29^, L. ch.^c^ , L. d.^20^ |
| Nigericin | 3.13 | 2.28 | Ionophore antibiotic ^18^ | L. d.^c^ ^18^ |

<sup>a<sup> EC_50_ = concentration of test compound that led to a 50% reduction in parasite counts in the ex vivo model.

<sup>b<sup> Determined by search of NCBI PubMed Database; in vitro activity in promastigotes unless stated otherwise. L.d.= Leishmania donovani; L. m.= Leishmania major; L. a.= Leishmania amazonensis; L. ch.= Leishmania chagasi; L.t.= Leishmania tarentolae; L. ae.= Leishmania aethiopica; L.p.= Leishmania panamensis; L. i.= Leishmania infantum; L.b.= Leishmania braziliensis.

<sup>c<sup> In vitro activity in amastigotes.

**References:**

1. Ephros M, Waldman E, Zilberstein D. 1997. Pentostam induces resistance to antimony and the preservative chlorocresol in Leishmania donovani promastigotes and axenically grown amastigotes. Antimicrob Agents Chemother. May;41(5):1064-8.

2. Roberts WL, Rainey PM. 1993. Antileishmanial activity of sodium stibogluconate fractions. Antimicrob Agents Chemother. Sep;37(9):1842-6.

3. Davidson RN, Dl Martino L, Artino L, Gradoni L, Giacchino R, Russo R, Gaeta GB, Pempinello R, Scott S, Raimondi F, Cascio A, Prestileo T, Caldeira L, Wilkinson RJ and Bryceson ADM. 1994. Liposomal amphotericin B (AmBisome) in Mediterranean visceral leishmaniasis: a multi-centre trial. Quarterly Journal of Medicine. 87:75-81

4. El-On J, Jacobs GP, Witztum and Greenblatt C. 1984. Development of Topical Treatment for Cutaneous Leishmaniasis Caused by Leishmania major in Experimental Animals. Antimicrob Agents Chemother. p. 745-751 Vol. 26, No. 5.

5. Sundar S, Chakravarty J, Rai VK, Agrawal N, Singh SP, Chauhan V, Murray HW. 2007. Amphotericin B treatment for Indian visceral leishmaniasis: response to 15 daily versus alternate-day infusions. Clin Infect Dis. Sep 1;45(5):556-61. Epub 2007 Jul 23.

6. Guimarães LH, Machado PR, Lago EL, Morgan DJ, Schriefer A, Bacellar O,

Carvalho EM. 2009. Atypical manifestations of tegumentary leishmaniasis in a transmission area of Leishmania braziliensis in the state of Bahia, Brazil. Trans R Soc Trop Med Hyg. Jul;103(7):712-5. Epub 2009 May 28.

7. Arana BA, Mendoza CE, Rizzo NR, Kroeger A. 2001. Randomized, controlled, double-blind trial of topical treatment of cutaneousleishmaniasis with paromomycin plus methylbenzethonium chloride ointment in Guatemala. Am J Trop Med Hyg. Nov;65(5):466-70.

8. Soto J, Fuya R, Herrera R, Berman J. 1998. Topical Paromomycin / methylbenzethonium chloride plus parenteral meglumine antimonate as treatment for American cutaneous leishmaniasis: controlled study. Clin Infect Dis 26: 56–58.

9. El-On J, Halevy S, Grunwald MH, Weinrauch L. 1992. Topical treatment of Old World cutaneous leishmaniasis caused by Leishmania major: a double-blind control study. J Am Acad Dermatol 27: 227–231.

10. Lal S, Thavundayil JX, Nair NP, Annable L, Ng Ying Kin NM, Gabriel A, Schwartz G. 2006. Levomepromazine versus chlorpromazine in treatment-resistant schizophrenia: a double-blind randomized trial. J Psychiatry Neurosci. Jul;31(4):271-9.

11. El-On J, Rubinstein N, Kernbaum S, Schnur LF. 1986. In vitro and in vivo anti-leishmanial activity of chlorpromazine alone and combined with N-meglumine antimonate. Ann Trop Med Parasitol. Oct;80(5):509-17.

12. Pearson RD, Manian AA, Hall D, Harcus JL, Hewlett EL. 1984. Antileishmanial activity of chlorpromazine. Antimicrob Agents Chemother. May;25(5):571-4.

13. Cipriani A, La Ferla T, Furukawa TA, Signoretti A, Nakagawa A, Churchill R,McGuire H, Barbui C. 2009. Sertraline versus other antidepressive agents for depression. Cochrane Database Syst Rev. Apr 15;(2):CD006117.

14. Gordon PR, Kerwin JP, Boesen KG, Senf J. 2006. Sertraline to treat hot flashes: a randomized controlled, double-blind, crossover trial in a general population. Menopause.Jul-Aug;13(4):568-75.

15. Pei JT, Shi ZH. 2008. An effective combined therapy for simple premature ejaculation. Zhonghua Nan Ke Xue. Aug;14(8):731-3.

16. Palit P, Ali NJ. 2008. Oral therapy with sertraline, a selective serotonin reuptake inhibitor, shows activity against Leishmania donovani. Antimicrob Chemother. May;61(5):1120-4. Epub 2008 Feb 13.

17.Glaser TA, Utz GL, Mukkada AJ. Mol Biochem Parasitol. 1992. Mar;51(1):9-15.The plasma membrane electrical gradient (membrane potential) in Leishmania donovani promastigotes and amastigotes.

18. Zilberstein D, Dwyer DM. 1984. Antidepressants cause lethal disruption of membrane function in the human protozoan parasite Leishmania. Science. Nov 23;226 (4677):977-9.

19. Antoine JC, Jouanne C, Ryter A, Benichou JC. 1988. Leishmania amazonensis: acidic organelles in amastigotes. Exp Parasitol. Dec;67(2):287-300.

20. Beraa T, Lakshmana K, Ghanteswaria D, Pala S, Sudhahara Islama MN, Bhuyana NR, Dasb P. 2005. Characterization of the redox components of transplasma membrane electron transport system from Leishmania donovani promastigotes. Biochimica et Biophysica Acta 1725, 314 – 326.

21. Brun R, Leon W. 1978. Effect of ethidium bromide on growth and morphology of Leishmania tarentolae promastigotes in vitro. Acta Trop. Sep;35(3):239-46.

22. Gray SJ, Ward TL, Southern LL, Ingram DR. 1998. Interactive effects of sodium bentonite and coccidiosis with monensin or salinomycin in chicks. Poult Sci. Apr;77(4):600-4.

23. Luque-Ortega JR, Saugar JM, Chiva C, Andreu D, Rivas L. 2003 Identification of new leishmanicidal peptide lead structures by automated real-time monitoring of changes in intracellular ATP. Biochem J. Oct 1;375(Pt 1):221-30.

24. Yip-Schneider MT, Nakshatri H, Sweeney CJ, Marshall MS, Wiebke EA, Schmidt CM. 2005. Parthenolide and sulindac cooperate to mediate growth suppression and inhibit the nuclear factor-kappa B pathway in pancreatic carcinoma cells. Mol Cancer Ther. Apr;4(4):587-94.

25. Tiuman TS, Ueda-Nakamura T, Garcia Cortez DA, Dias Filho BP, Morgado-Díaz JA, de Souza W, Nakamura CV. 2005. Antileishmanial activity of parthenolide, a sesquiterpene lactone isolated from Tanacetum parthenium. Antimicrob Agents Chemother. Jan;49(1):176-82.

26. Vieira AT, Pinho V, Lepsch LB, Scavone C, Ribeiro IM, Tomassini T, Ribeiro-dos-Santos R, Soares MB, Teixeira MM, Souza DG. 2005. Mechanisms of the anti-inflammatory effects of the natural secosteroids physalins in a model of intestinal ischaemia and reperfusion injury. Br J Pharmacol. Sep;146(2):244-51.

27. Guimarães ET, Lima MS, Santos LA, Ribeiro IM, Tomassini TB, Ribeiro dos Santos R, dos Santos WL, Soares MB. 2009. Activity of physalins purified from Physalis angulata in in vitro and in vivo models of cutaneous leishmaniasis. J Antimicrob Chemother. Jul;64(1):84-7. Epub May 19.

28. Brennan J, Bagg R, Barnum D, Wilson J, Dick P. 2001. Efficacy of narasin in the prevention of necrotic enteritis in broiler chickens. Avian Dis. Jan-Mar;45(1):210-4.

29. Lemesre JL, Sereno D, Daulouède S, Veyret B, Brajon N, Vincendeau P. 1997. Leishmania spp.: nitric oxide-mediated metabolic inhibition of promastigote and axenically grown amastigote forms. Exp Parasitol. May;86(1):58-68.
